# Patient-reported Outcomes in Facial Reconstruction: Assessment of FACE-Q Scales and Predictors of Satisfaction

**DOI:** 10.1097/GOX.0000000000002004

**Published:** 2018-12-05

**Authors:** Adekunle Elegbede, Sara Mermulla, Silviu C. Diaconu, Colton McNichols, Yuanyuan Liang, Fan Liang, Yvonne M. Rasko, Michael P. Grant, Arthur J. Nam

**Affiliations:** From the * Department of Plastic and Reconstructive Surgery, The Johns Hopkins Hospital, Baltimore, Md; † Department of Surgery, University of Maryland School of Medicine Baltimore, Md; ‡ Department of Epidemiology and Public Health, University of Maryland School of Medicine, Baltimore, Md; § Department of Surgery, Division of Plastic, Reconstructive, and Maxillofacial Surgery, R Adams Cowley Shock Trauma Center, Baltimore, Md; ¶ Department of Surgery, Division of Plastic and Reconstructive Surgery, University of Maryland School of Medicine, Baltimore, Md.

## Abstract

**Background:**

There is a paucity of patient-reported outcome measures for facial trauma reconstruction. To measure satisfaction and health-related quality of life (HRQOL), following repair of traumatic facial fractures, we used the FACE-Q, a set of patient-reported outcome instruments designed for aesthetic facial surgery. As a step toward validating the scales for facial trauma, we evaluated their reliability.

**Methods:**

This is a prospective study of patients following primary repair of traumatic facial fractures at a level 1 trauma center from 2016 to 2018. Six FACE-Q scales with relevance to the facial trauma population were completed by patients at their 1-month postoperative visits. Predictors of satisfaction were examined using multiple linear regression models. Reliability of the scales in this population was evaluated using psychometric methods.

**Results:**

One hundred eighty-five participants fulfilled inclusion criteria. Mean scores for the 6 scales ranged from 59 (SD = 15) for Recovery-Early Life Impact to 94 (SD = 13) for *Satisfaction with Medical Team*. Predictors of lower satisfaction and/or HRQOL include current tobacco smoking status, mandibulomaxillary fixation, and Le Fort pattern fractures. All scales were found to have good to excellent reliability (Cronbach’s alpha = 0.824–0.969).

**Conclusions:**

Following repair of facial fractures, patient-reported outcomes can be reliably measured using FACE-Q scales. On average, patients report poor health-related quality of life in the early postoperative period. Predictors of low satisfaction and/or poor HRQOL include current smoking habit, mandibulomaxillary fixation, and Le Fort fractures.

## INTRODUCTION

Facial trauma impacts victims across several quality of life domains. Patients may suffer facial disfigurement, reduced self-esteem, psychosocial distress, and difficulty with activities of daily living.^1–3^ Furthermore, aspects of surgical treatment may negatively impact the patient. In the past few decades, patient-reported outcomes have been increasingly utilized to assess the benefits of cosmetic procedures, advocate for access to reconstructive procedures, compare surgical techniques, and provide quality metrics that may stimulate improvement in care delivery.^4,5^ When the condition of interest is limited to an anatomic region, a condition-specific instrument may be more relevant and responsive to differences in the patients’ perception of outcomes.^6^

A number of scales have been validated for measuring patient perceptions of facial disfigurement or quality of life following facial paralysis.^3^ Other scales, such as the FACE-Q have been utilized for assessing satisfaction following facial aesthetic procedures.^7–11^ Although use of FACE-Q has thus far been restricted to the domain of cosmetic surgery, it serves to reason that it may also be applicable to the reconstructive trauma population, given the significant overlap between goals of aesthetic and reconstructive surgery.^12^ The purpose of this study was to (1) using FACE-Q scales, measure patient-reported outcomes following facial fracture reconstruction; (2) identify any factors affecting these outcomes; and (3) demonstrate the reliability of the FACE-Q scales as a step toward its validation in the facial trauma population.

## METHODS

This is a prospective study conducted from 2016 to 2018 by the Division of Plastic, Reconstructive and Maxillofacial Surgery at R Adams Cowley Shock Trauma Center of the University of Maryland Medical Center, a Level 1 trauma center. Institutional review board approval (#HP-00066231) was obtained before beginning the study.

### Study Sample

We enrolled patients in our clinic who presented for postoperative evaluation following primary reconstruction of facial fractures that were sustained from trauma. We excluded patients who were unable to read English, did not undergo surgical repair of facial fractures, had pathologic fractures, or sustained traumatic brain injury. The goal sample size of 200 participants was based on our estimated volume of approximately 100 unique patients per year who meet inclusion criteria, carried out over a 2-year enrollment period.

### Instruments and Data Collection

Six scales were selected from the 40-plus available FACE-Q scales, based on relevance to the facial trauma population.^9,10^ Each scale is scored ranging from 0 (lowest) to 100 (highest). The items comprising each scale and the corresponding satisfaction/QOL domains have been described in detail previously.^9,13^ Briefly, the 6 scales that we utilized are as follows:

1. *Satisfaction with Facial Appearance Overall*: An appearance appraisal scale that evaluates the face as a whole. There are 10 items that assess self-perceptions of facial symmetry, proportion, and how the face appears at different times and under varying conditions.
2. *Recovery-Early Life Impact*: This scale includes 12 items designed to assess comfort and the capacity to perform normal basic living activities in the early recovery phase. In addition, this scale evaluates feelings of anxiety and embarrassment in social situations, during the recovery period.
3. *Social Function*: This scale has 8 items that are designed to measure the quality of social interactions, including those with friends and strangers. It also evaluates one’s confidence in making a positive first impression.
4. *Psychological Well-Being*: This scale has 10 items that measure psychosocial functioning, such as happiness, believing in one’s self, confidence and the feeling of being in control.
5. *Satisfaction with Outcome*: This scale had 6 items that measure overall patient satisfaction with the result of his/her most recent procedure.
6. *Satisfaction with Medical Team*: This scale measures patient satisfaction with members of the medical team, excluding the surgeon. The 10 items in this scale determine satisfaction with privacy, staff friendliness, attentiveness, and knowledge.

All surveys were given to patients during their 1 month postoperative clinic visits. To reduce the potential influence of the medical team members on survey responses, the surveys were presented by a research fellow who is not involved in patient care. The raw scores from the survey were entered into an electronic database and the corresponding scaled scores recorded.

### Clinical Variables of Interest

Patient demographics including age, sex, race, and smoking status were recorded. Injury and treatment variables were also recorded.

### Data Analyses/Statistics

Clinical variables and Rasch scores for each scale were summarized using counts and percentages for categorical variables. For continuous variables, the mean ± SD or the median, lower (Q1) and upper quartiles (Q3) were used.

Multiple linear regression models were utilized to identify variables that are predictive of patient satisfaction or health-related quality of life (HRQOL). For each outcome of interest, a backward model selection procedure was used. Potential predictors examined in the full model included race, age, sex, smoking status, body mass index (BMI), mechanism of injury, fracture location, fracture pattern, mandibulomaxillary fixation (MMF), and duration between surgery and survey completion. Predictors with *P* < 0.1 in the full model were included in the final reduced regression model. All statistical tests were conducted at a 2-sided significance level of 5%, and all analyses were performed using Stata/SE (version 14, College Station, Tex.). Reliability of the scales (ie, the degree to which it is free from random error) was evaluated by means of Cronbach’s α. Internal consistency is commonly described as excellent when α ≥ 0.9, good when α is between 0.8 and 0.9, and acceptable when α is between 0.7 and 0.8. α < 0.7 Implies that internal consistency is questionable, poor, or unacceptable.

## RESULTS

### Study Sample

One hundred ninety-one of the 194 patients who were presented with surveys completed the surveys. Three patients declined to participate. Six completed surveys were further excluded because the patients were undergoing secondary or tertiary repair for fractures that had been treated elsewhere, resulting in a total of 185 participants for the data analysis. Summary of demographic/baseline data is presented in Table 1. Participants were predominantly male, in their mid-twenties to mid-forties, and were current or former smokers. Median days from surgery to survey completion was 29 days (Q1 = 15, Q3 = 51). The locations and patterns of the fractures sustained by participants are summarized in Table 2. Mid and lower face fractures were most frequent, reflective of the distribution of facial fracture repairs at our institution.

**Table 1.**
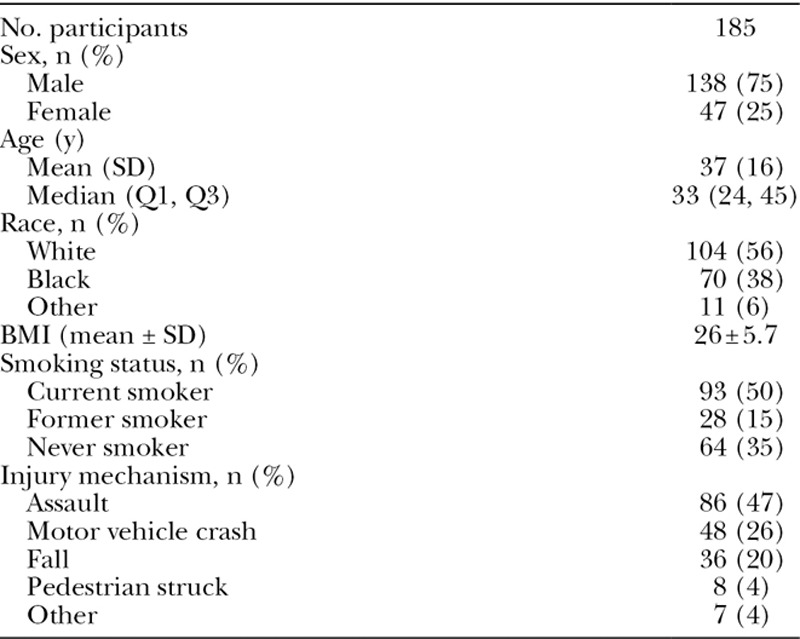
Demographics and Baseline Data

**Table 2.**
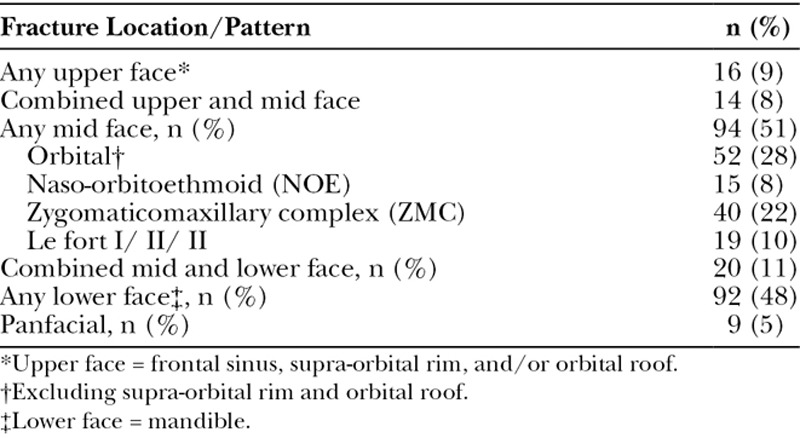
Fracture Characteristics

### Satisfaction and Quality of Life Scores

Mean scores for the 6 scales ranged from 59 for *Recovery-Early Life Impact* to 94 for the *Satisfaction with Medical Team* surveys (Table 3).

**Table 3.**
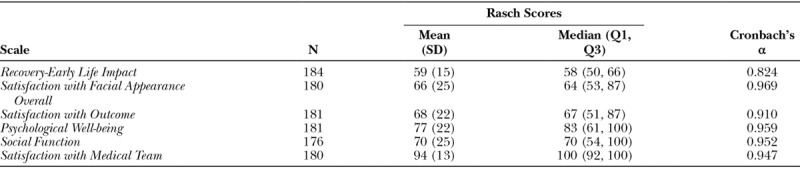
Summary of Scores and Cronbach’s Alpha for the 6 Scales

### Reliability of the Instrument and Dataset

Internal consistency was good (Cronbach’s alpha: 0.824) for the *Recovery-Early Life Impact* scale and excellent (Cronbach’s alpha > 0.900) for all of the other scales (Table 3).

### Predictors of Satisfaction and Quality of Life

Predictor variables identified by the full regression models are shown in Table 4 for each of the 6 scales. Smoking status, MMF, race, and duration from surgery to survey emerged as predictors for at least 2 of the 6 scales. Injury mechanism, orbit fractures, upper face fractures, and Lefort pattern fractures emerged as predictors for 1 of the 6 scales. No single variable was predictive of outcome across all 6 scales. Age, sex, BMI were not identified as significant predictors on any of the 6 scales. Of note, “Other” race or “Other” injury mechanism were identified as significant predictors on 3 of the scales and merit further description: The 11 patients classified as “Other” race included 3 patients of Asian descent, 3 patients of Middle Eastern descent, and 5 Hispanic patients that do not identify as White. The 7 patients with injury mechanism of “Other” included 3 mechanical work-related injuries (eg, struck by forklift), 2 self-inflicted gunshots, and 2 dog bites.

**Table 4.**
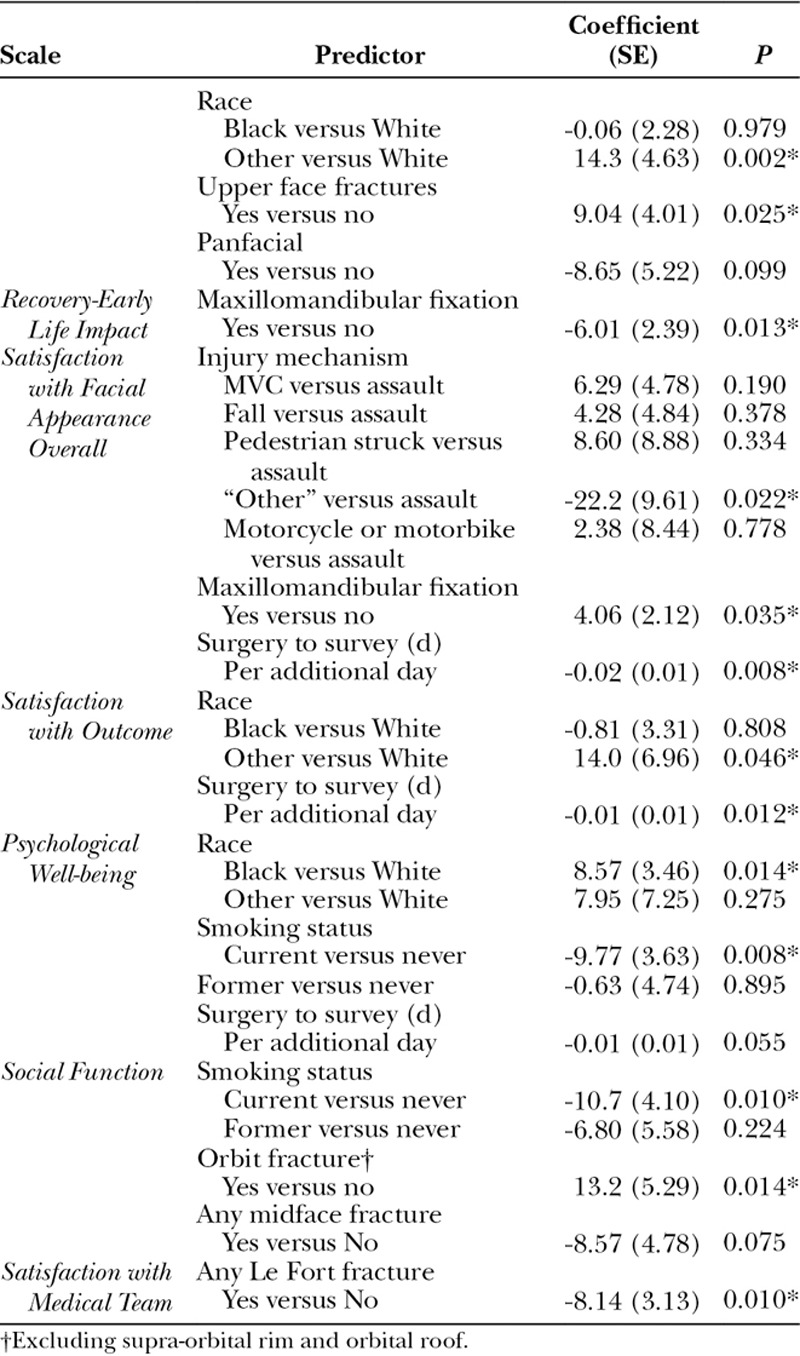
Multiple Linear Regression Predictors for the 6 Scales

## DISCUSSION

The key findings from this study are as follows: (1) Following repair of traumatic facial fractures, patient satisfaction/ HRQOL varies across domains: *Satisfaction with the medical team* is high, whereas *Recovery-Early Life Impact* is relatively poor; (2) Factors predictive of low satisfaction and/or HRQOL include: current smoking habit, MMF, and Le Fort fractures. (3) FACE-Q scales demonstrate good to excellent reliability in this population.

Although there are no published normative values for FACE-Q, subgroup differences that are greater than one-half the SD are considered clinically meaningful.^6,14^ Furthermore, scores from published studies may provide clinical context for interpreting our scores. In the following paragraphs, we compare the scores (obtained postoperatively) on the 6 scales, with scores from published studies.

For the *Recovery-Early Life Impact* scale, mean scores of 80 to 93 were observed in 2 studies 1 month after aesthetic facial surgery/rejuvenation.^10,15^ Our lower mean score of 59 is not surprising. Given that facial fracture repair is generally more invasive than most aesthetic facial procedures, it should be expected to more severely impact daily activities in the early recovery period. Furthermore, many of our study participants sustained polytrauma and may have been experiencing the sequelae of nonfacial trauma.

For the *Satisfaction with Facial Appearance* scale, our mean score of 66 compares with mean preprocedure scores of 45 to 53, and postprocedure scores of 64 to 87 for patients undergoing a variety of aesthetic procedures.^16,17^ Of note, the patient population in these aesthetic studies is older and predominantly female.^8,18^ Nonetheless, it is noteworthy that facial trauma patients, many of whom have significant facial disfigurement, report higher satisfaction with facial appearance when compared with patients seeking facial aesthetic surgery.^19^ The composition of the “Other” injury mechanism group (gunshots, dogbites, forklift trauma) likely explains their lower scores. It is unclear why longer duration from surgery is associated with lower scores on this scale. A possible explanation is that unsatisfied patients are more likely to return for postoperative visits, whereas satisfied patients may be lost to follow-up.

Our *Social Function* scores are similar to published scores from the aesthetic surgery population.^15,20–22^ The association of cigarette smoking habit with lower social function is consistent with known associations of smoking and social isolation.^23^ However, to our knowledge, this relationship has not been previously demonstrated in the context of facial aesthetic or reconstructive surgery.

Published averages for the *Psychological* Well-being scale range from 69, 1 month after aesthetic facial surgery/nonsurgical rejuvenation to 93, after facelifts.^15,20–22^ Our scores (mean = 77, Q1 = 61) are high, given elevated rates of post-traumatic stress disorder (PTSD) and mental disorders following facial trauma.^24,25^ Our findings of worse psychological well-being among cigarette smokers and better psychological well-being among patients of black race are consistent with known associations of smoking habit^26–28^ and race^29^ with psychological health.

We found no published scores for the *Satisfaction with Medical team* scale. It is unclear why patients with Le Fort fractures would score lower on this scale. Nevertheless, facial trauma teams should be attentive to the potential for dissatisfaction with the process of care among these patients.

Our internal consistency results compare favorably with published scores from the aesthetic surgery population. The relatively low value Cronbach’s α for the *Recovery-Early Life Impact* scale was also observed in the aesthetic surgery population.^10^

The strengths of our study are as follows: This is the first study that seeks to validate FACE-Q scales in the facial trauma population. Utilizing FACE-Q scales may obviate the need to develop separate patient-reported outcome instruments for facial trauma. Second, the number of participants and the response rate for this study are high, affording high reliability and statistical power. Third, the prospective study design should minimize errors due to recall bias, or bias in scoring due to temporal variations in satisfaction or quality of life.

This study has several limitations: First, scientific soundness of the scales in the facial trauma population has not been fully established. In addition to reliability which we have shown, validity and responsiveness need to be demonstrated in the target population. Second, we do not have presurveys that would permit us to adjust for preinjury or preoperative factors. Obviously, it is impractical to collect preinjury data. For most of the patients at our center, surgery is performed within hours to days of injury, during which patients may be under distress that may diminish responsiveness and reliability of the data. Third, the location, severity, soft tissue involvement and surgical procedure that participants underwent are variable. Finally, our regression models did not account for factors like psychological history, employment, family support, litigation, incarceration, which have been shown to affect quality of life following facial trauma.^24^

## CONCLUSIONS

Face-Q scales can be utilized for measuring satisfaction and patient perception following repair of traumatic facial fractures. Excellent response rates can be achieved. The scales demonstrate good to excellent reliability in this population. Participants report high satisfaction with the medical team, but poor health-related quality of life in the early postoperative period. Predictors of low satisfaction and/or poor HRQOL include current smoking habit, MMF, and Le Fort fractures.
